# Using data linkage for national surveillance of clinical quality indicators for dementia care among Australian aged care users

**DOI:** 10.1038/s41598-021-89646-x

**Published:** 2021-05-21

**Authors:** Monica Cations, Catherine Lang, Stephanie A. Ward, Gillian E. Caughey, Maria Crotty, Craig Whitehead, Susannah Ahern, John Maddison, Maria C. Inacio

**Affiliations:** 1 Registry of Senior Australians, South Australian Health and Medical Research Institute, PO Box 11060, Adelaide, SA 5001 Australia; 2 College of Medicine and Public Health, Flinders University, Adelaide, SA Australia; 3 Centre for Healthy Brain Ageing, University of New South Wales, Sydney, NSW Australia; 4 Department of Geriatric Medicine, The Prince of Wales Hospital, Sydney, NSW Australia; 5 School of Public Health and Preventative Medicine, Monash University, Melbourne, VIC Australia; 6 Faculty of Health and Medical Sciences, University of Adelaide, Adelaide, SA Australia; 7 Division of Health Sciences, University of South Australia, Adelaide, SA Australia; 8 Southern Adelaide Local Health Network, SA Health, Adelaide, SA Australia; 9 Northern Adelaide Local Health Network, SA Health, Adelaide, SA Australia

**Keywords:** Health services, Epidemiology, Dementia

## Abstract

Care quality has important implications for people with dementia. We examined trends and geographical variation of four clinical quality indicators (CQIs) in Australia. This retrospective cohort study included all people with dementia using Australian government-subsidised aged care in 2008–2016 (n = 373,695). Quality indicator data were derived from linked national aged care, health, and pharmaceutical datasets. Negative binomial regression modelling assessed trends in CQI performance over time (2011–2016) and funnel plots examined geographical variation in performance. The incidence rate of antipsychotic medicine dispensing decreased slightly from 1.17/1000 person-days to 1.07/1000 person-days (adjusted incidence rate ratio (aIRR) = 0.98, 95%CI 0.98–0.99). Cholinesterase inhibitors and memantine dispensing did not change (aIRR = 1.02, 95%CI 1.00–1.04), while exposure to high sedative load increased slightly from 1.39/1000 person-days to 1.44/1000 person-days (aIRR = 1.01, 95%CI 1.00–1.01). Dementia and delirium-related hospitalisations increased slightly from 0.17/1000 person-days to 0.18/1000 person-days (aIRR = 1.02, 95%CI 1.01–1.03). There was marked variation in cholinesterase inhibitor and memantine dispensing by geographical area (0–41%). There has been little change in four indicators of dementia care quality in Australian aged care users over time. Cholinesterase inhibitor and memantine dispensing varied substantially by geographical region. Existing strategies to improve national performance on these indicators appear to be insufficient, despite the significant impact of these indicators on outcomes for people with dementia.

## Introduction

The quality and safety of care that people with dementia receive has an important impact on their quality of life, symptom progression, and mortality^1–3^. As the global prevalence of dementia continues to rise, advocacy bodies including the World Health Organization (WHO) have emphasised the need for increasing investment in policies, programs, and interventions that improve the quality of dementia care^4^. However, monitoring care quality can be difficult because dementia care occurs in a variety of contexts and settings, and involves a wide range of professionals. Clinical quality indicators (CQIs) are tools used across health and aged care systems as population-level measures of care quality and safety^5^. CQIs are measurable structures, processes, or outcomes that can be used to monitor and benchmark the care provided to people with dementia. Variations in CQI performance can indicate geographical areas or populations most in need for policy and quality improvement intervention^6^.

Priority CQIs for dementia diagnosis^7, 8^, care^9^, management of behavioural symptoms^10^, and palliative care^11^ have been identified in earlier studies. While dementia registries can facilitate monitoring and benchmarking of CQIs, some of these registries are a relatively new and are currently limited to a cluster of Scandinavian countries^12^. Similar registries are being established internationally including in Australia, but are not yet able to monitor care quality^13^.

In Australia, approximately 47% of people living in residential aged care and 21% of people accessing long-term home care services have a recorded diagnosis of dementia^14^, representing 40% (*n* = 135,555) of the estimated prevalent dementia cases in Australia^15^. Routinely-collected aged and health care records can make an important contribution to CQI monitoring especially in countries like Australia with universal health care and strong social welfare systems. The Registry of Senior Australians (ROSA) platform, which has linked the aged care and health care records of older people receiving aged care services in all Australian states and territories, can be used to derive CQIs that can be used to monitor the post-diagnosis care quality for the large number of people with dementia in aged care settings. In ROSA, individual de-identified data from aged care eligibility and funding assessments is linked to data about health service use, pharmaceutical use, hospitalisations, and death. This data linkage is periodically updated and facilitates monitoring of service use and examination of the longitudinal impacts of this use without the need for additional data collection^16, 17^.

The aim of this study is to (a) examine the performance of four selected CQIs for dementia care over time in aged care users, (b) assess demographic factors associated with CQI variation, and (c) examine the geographic variation in CQI performance. Four CQIs were evaluated: exposure to antipsychotic medicines, use of cholinesterase inhibitors and memantine, exposure to high sedative medicine load, and dementia or delirium-related hospitalisations. While not an exhaustive list, these CQIs have been identified as important indicators of care quality in international Delphi studies^8, 9^ because of their significant impact on quality of life and wellbeing. Antipsychotic medicines are commonly used in Australia^18^ and internationally^19^ to treat behavioural and psychological symptoms of dementia (BPSD) but are known to have limited efficacy^20^ and harmful side effects^21^. Similarly, people with dementia are highly susceptible to adverse drug events (ADEs) from sedating medicines including anxiolytics and antidepressants^22^. A wide range of efforts have therefore been implemented that aim to reduce the prescribing of antipsychotic and other sedative medications^23^. Hospitalisations related to dementia or delirium can indicate potentially preventable complications and are associated with poorer outcomes including mortality risk^24^.

Finally, clinical guidelines^25, 26^ recommend trial of a cholinesterase inhibiting medicine or memantine for all people with mild to moderately-severe Alzheimer’s disease (AD) because these medicines can slow the progression of cognitive impairment in some cases^27^. However, views vary about the utility of these medicines among prescribers and this affects prescribing behaviour^28, 29^ and there is an associated need to increase prescribing^25^. These four CQIs are therefore highly relevant indicators of dementia care quality, and all can be monitored using routinely-collected administrative data^7, 8^.

## Method

### Design and participants

A retrospective cohort and cross-sectional evaluation using the ROSA National Historical Cohort was conducted. The ROSA National Historical Cohort includes de-identified linked aged care and health care service records for older Australians who accessed government-subsidised aged care services from 1997, including home care packages, permanent residential aged care, transition care, and respite care^16^. Data used for this study included demographic and clinical data from aged care eligibility and entry into care assessments captured in the publicly available National Aged Care Data Clearinghouse and maintained by the Australia Institute of Health and Welfare. These data were linked to medicine dispensing records from the Pharmaceutical Benefits Scheme (PBS), and state health authorities provided hospitalisation records (for South Australia, New South Wales, and Victoria only). Date of death was identified from the National Death Index and was used to identify the individual’s date of exit from the cohort. Linkage for ROSA was conducted by the data custodian, the Australian Institute of Health and Welfare, with governance approval. More detail about data linkage procedures for ROSA have been previously published^16^.

People with a diagnosis of dementia recorded on aged care assessments or who have been dispensed an anti-dementia medicine (donepezil, galantamine, rivastigmine, memantine, see Supplementary Table A1) were included in the study cohort. Assessments are conducted to determine eligibility for aged care services under the Aged Care Assessment Program^30^, or to determine levels of funding needed to support a person in permanent residential care using the Aged Care Funding Instrument^31^. Assessors are required to provide evidence for the dementia diagnosis by a health care professional.

The date of cohort entry in ROSA is the first aged care assessment. Date of entry to this study is the date of the first aged care assessment where dementia was recorded; this may be later than the date of entry to the ROSA cohort because the person may have had an earlier aged care assessment where dementia was not recorded. Both the date of cohort entry and date of study entry are distinct from the date of dementia diagnosis, which is unknown in this cohort. Future linkage of ROSA data with the Australian Dementia Registry CQR currently in development is planned and will provide more information about symptom onset and diagnosis. However, these data were not available for the current study. More details about people with dementia in ROSA have been previously published^17^.

All non-Indigenous Australians aged 65 years or older who were identified with a diagnosis of dementia between 1 July 2008 and 30 June 2016 were included in this study (*n* = 373,695).

### Measures

#### Clinical quality indicators (CQIs)

Four CQIs were examined: antipsychotic medicine dispensing, anti-dementia medicine dispensing, high sedative load, and dementia and delirium-related hospitalisations (South Australia, New South Wales, Victoria only). Trends in these CQIs were examined for the period of 2011 to 2016; a three-year lead in time was considered sufficient to accurately capture the total population. Antipsychotic medicine exposure was defined as at least one antipsychotic medicine dispensing for each financial year over the study period (i.e. between 2011/2012 and 2015/16). Number of days exposed to antipsychotics was also estimated by dividing the quantity supplied at each dispensing by the usual number of doses per day in older people from Australian prescribing guidelines^32^. Cholinesterase inhibitor or memantine dispensing was present where an individual was dispensed donepezil, galantamine, rivastigmine or memantine at least once in the financial year of interest. Given the different indications, biological mechanisms, and clinical uses between memantine and cholinesterase inhibitors, we conducted a sensitivity analyses where memantine was excluded^27^.

Antipsychotic medicines and cholinesterase inhibitors included in the study are described in Supplementary Table A1 and were coded in the dataset according to the WHO Anatomical and Therapeutic Chemical (ATC) classification system.

Sedative medicine load was calculated using the rating system developed by Linjakumpu and colleagues^33^, whereby a high sedative load is defined as a score of ≥ 3 during any 90-day period within the given year. Dementia or delirium-related hospitalisations were ascertained from hospital or emergency department admissions with the principal discharge diagnosis of dementia or delirium. Hospitalisations are classified according to the International Classification of Diseases, 10th Revision, Australian Modification and the codes used to identify these hospitalisations are described in Supplementary Table A1. Hospitalisation records were only available for the states of South Australia, New South Wales, and Victoria (68.3% of the cohort).

#### Factors associated with CQI performance

Age, sex, and country of birth (Australia vs other), number of co-morbid conditions, geographical region, and state were examined as potential factors contributing to the trends in CQI. These factors were chosen based on evidence that they impact on the quality of care a person with dementia will receive^34^. This is not an exhaustive list however and many variables not available in the ROSA dataset may also have an impact; this is discussed in more detail in the Limitations section below. Number of comorbid health conditions were determined using the RxRisk-V^35^, a medicine-based comorbidity index based on six months history of medicine dispensing prior cohort entry. Location at time of aged care eligibility was classified using the Accessibility/Remoteness Index of Australia Plus (ARIA+)^36^ and categorised as major city vs regional/remote. State was determined by postcode at the time of assessment or at entry to residential care. New South Wales was used as the reference category because it is the most populous Australian state (34% of cohort).

#### Denominator

Cohort person-days for each study year was used as the denominator in all analyses. Person-days was determined by calculating the number of days each individual was alive in the cohort during that year. Those who entered the cohort after the given year or died before the year were not included in calculation of person-days for that year.

### Analysis

The incidence rate and 95% confidence interval (CI) of each CQI was calculated per financial year, using the number of person-days in each year as the denominator. Changes in the incidence of each CQI over the study period, adjusted for age and sex, were examined with year as the exposure variable and CQI performance as the outcome variable. We also examined factors associated with CQI performance for the 2015/16 financial year. As cholinesterase inhibitors and memantine are indicated for mild- to moderately-severe AD only, all analysis of this CQI was adjusted for time since cohort entry (as a proxy measure of time since symptom onset). Negative binomial regression modelling was used because of overdispersion in the data distribution. Incidence rate ratios and 95% CIs are reported. Generalised linear modelling was used to assess changes in the proportion of days exposed to antipsychotic medicines over time. All tests were two-sided and adjusted for multiple hypothesis comparisons using the stepdown Bonferroni method, and α < 0.0125 was considered statistically significant in final models. Missing data (< 2.1%) were managed via case wise deletion.

Incidence rates were calculated by state to assess geographical variation in indicator performance. In addition, geographical variations were examined graphically using funnel plots adjusted for age and sex. Variation is plotted using statistical area (SA)-3 regions, geographical areas built by the Australian Bureau of Statistics with populations between 30,000 and 130,000 persons^37^. The Wilson method for binomially distributed estimates was used to calculate 95% and 99.8% CIs around the population mean^38^. Data were analysed using SAS Software version 9.4 (SAS Institute Inc., Cary, NC, USA)^39^.

### Ethics

Ethical approval for this study was provided by the University of South Australia (ID200489), Australian Institute of Health and Welfare (EO2018/1/418), NSW Population and Health Services Research Ethics Committee (2019/ETH12028), and the South Australian Department for Health and Wellbeing (HREC/18/SAH/90) Human Research Ethics Committees. All methods were performed in accordance with the Australian National Health and Medical Research Council’s National Statement on Ethical Conduct in Human Research^40^. In accordance with this statement, informed consent from individual participants was not required because data used here is de-identified and publicly available.

## Results

There were 373,695 people identified with dementia in the ROSA cohort in the study period (Table 1). They were aged 84.1 years on average at cohort entry (SD = 6.9 years) and 63.1% were female. Nearly 33% were born outside Australia, and 11.5% spoke a primary language other than English.

**Table 1:** Overall study cohort demographic characteristics and co-morbidity burden (*n* = 373,695).

	Missing *n* (%)	ẋ (SD) or *n* (%)
Age at first dementia identification (in assessment data)	0	84.1 (6.9)
Female	49 (0.1)	235,703 (63.1)
Born outside Australia	2290 (0.6)	123,009 (32.9)
Primary language other than English	1808 (0.5)	42,779 (11.5)
**State**	4754 (1.3)	
Australian Capital Territory		4114 (1.1)
New South Wales		127,830 (34.2)
Northern Territory		780 (0.2)
Queensland		63,986 (17.1)
South Australia		34,252 (9.2)
Tasmania		9379 (2.5)
Victoria		92,911 (24.9)
Western Australia		35,689 (9.6)
**ARIA + region**	7533 (2.0)	
Major city		245,089 (65.6)
Inner regional		78,320 (21.0)
Outer regional		37,378 (10.0)
Remote		4074 (1.1)
Very remote		1307 (0.4)
**Socio-economic status (measured using IRSD)**	7600 (2.0)	
Most Disadvantaged -1		61,573 (16.5)
2		63,830 (17.1)
3		65,613 (17.6)
4		70,050 (18.8)
Least Disadvantaged- 5		105,029 (28.1)
**Number of comorbid conditions**	25,875 (6.9)	
0		11,862 (3.2)
1–4		170,826 (45.7)
5–9		151,358 (40.5)
10 +		13,747 (3.7)
Days of follow up (median, IQR)	0 (0)	688 (282–1300)
Deceased at June 30 2016	0 (0)	249,682 (66.8)

*ARIA* Accessibility/Remoteness Index of Australia, *IQR* Interquartile range, *IRSD* Index of Relative Socio-economic disadvantage, *SD* Standard deviation.

### Trends over time

The incidence rate for antipsychotic medicine dispensing decreased from 1.17 (95%CI: 1.16–1.18) per 1000 person-days in 2011/12 to 1.07 (95%CI: 1.06–1.08) per 1000 person-days in 2015/16 (aIRR = 0.98, 95%CI 0.98–0.99, *p* < 0.001; Table 2). There was also a slight reduction in the total proportion of days exposed to antipsychotics after adjusting for age and sex (23.1% in 2011/12 to 21.5% in 2015/16, *p* < 0.001). The incidence rate for anti-dementia medicine dispensing increased from 0.62 (95%CI: 0.62–0.63) per 1000 person-days in 2011/12 to 0.65 (95%CI: 0.65–0.66) in 2015/16, but this change was not statistically significant (aIRR = 1.02, 95%CI: 1.00–1.04, *p* = 0.05). This was also true after removal of memantine from the analysis (aIRR = 1.01, 95%CI: 0.99–1.04, *p* = 0.16; Supplementary Table A2).

**Table 2:** Incidence rate and trends in yearly clinical quality indicator performance.

	2011/12	2012/13	2013/14	2014/15	2015/16	Absolute change ^a^	aIRR (95%CI) ^b^	*p* ^c^
Yearly denominator (*n*)	153,575	158,041	161,306	163,948	159,378			
Person-days (*n*)	43,178,253	44,459,569	45,634,657	46,272,751	46,510,686			
Antipsychotic medicine dispensing (*n*)	50,315	50,691	51,423	51,572	49,920			
Cumulative incidence (95% CI)	32.8 (32.5–33.0)	32.1 (31.8–32.3)	31.9 (31.7–32.1)	31.5 (31.2–31.7)	31.3 (31.1–31.6)	− 1.5		
Incidence per 1000p/d (95% CI)	1.17 (1.16–1.18)	1.14 (1.13–1.15)	1.13 (1.12–1.14)	1.11 (1.10–1.12)	1.07 (1.06–1.08)	− 0.10	0.98 (0.98–0.99)	< 0.001
Cholinesterase inhibitor and memantine dispensing (*n*)	26,926	28,102	29,637	31,281	30,376			
Cumulative incidence (95% CI)	17.5 (17.3–17.7)	17.8 (17.6–18.0)	18.4 (18.2–18.6)	19.1 (18.9–19.3)	19.1 (18.9–19.3)	1.6		
Incidence per 1000p/d (95% CI)	0.62 (0.62–0.63)	0.63 (0.62–0.64)	0.65 (0.64–0.66)	0.68 (0.67–0.68)	0.65 (0.65–0.66)	0.03	1.02 (1.00–1.04) ^d^	0.05
High sedative load (*n*)	60,096	63,315	65,741	66,743	67,074			
Cumulative incidence (95% CI)	39.1 (38.9–39.4)	40.1 (39.8–40.3)	40.8 (40.5–41.0)	40.7 (40.5–41.0)	42.1 (41.8–42.3)	3.0		
Incidence per 1000p/d (95% CI)	1.39 (1.38–1.40)	1.42 (1.41–1.44)	1.44 (1.43–1.45)	1.44 (1.43–1.45)	1.44 (1.43–1.45)	0.05	1.01 (1.00–1.01)	< 0.001
Dementia or delirium-related hospitalisation (*n*) ^e^	4873	5103	5428	5691	5578			
Denominator	105,117	107,638	109,758	111,179	107,804			
Person-days (n)	29,541,548	30,287,420	31,014,578	31,323,189	31,340,451			
Cumulative incidence (95% CI)	4.6 (4.5–4.8)	4.7 (4.6–4.9)	4.9 (4.8–5.1)	5.1 (5.0–5.3)	5.1 (5.0–5.3)	0.5		
Incidence per 1000p/d (95%CI)	0.17 (0.16–0.17)	0.17 (0.16–0.17)	0.18 (0.17–0.18)	0.18 (0.18–0.19)	0.18 (0.17–0.18)	0.01	1.02 (1.01–1.03)	0.001

*aIRR* Adjusted incidence rate ratio, *CI* Confidence interval; *p/d* Person-days.

^a^From 2011/2012 to 2015/2016—crude difference only.

^b^Annual change adjusted for age (at beginning of each year) and sex.

^c^Bonferroni correction applied.

^d^Adjusted for age (at beginning of each year), sex, and time since cohort entry.

^e^New South Wales, Victoria, and South Australia only.

There was a small increase in the incidence rate of exposure to high sedative load over time, from 1.39 per 1000 person-days in 2011/12 to 1.44 per 1000 person-days in 2015/16 (aIRR = 1.01, 95%CI: 1.00–1.01, *p* < 0.001). There were 254,993 people living in New South Wales, Victoria, or South Australia included in analysis of the incidence rate of dementia and delirium-related hospitalisations, which also increased from 0.17 (95%CI: 0.16–0.17) per 1000 person-days in 2011/12 to 0.18 (95%CI: 0.17–0.18) per 1000 person-days in 2015/16 (aIRR = 1.02, 95%CI: 1.01–1.03, *p* < 0.001).

### Variation in CQI performance

**Figure 1:**
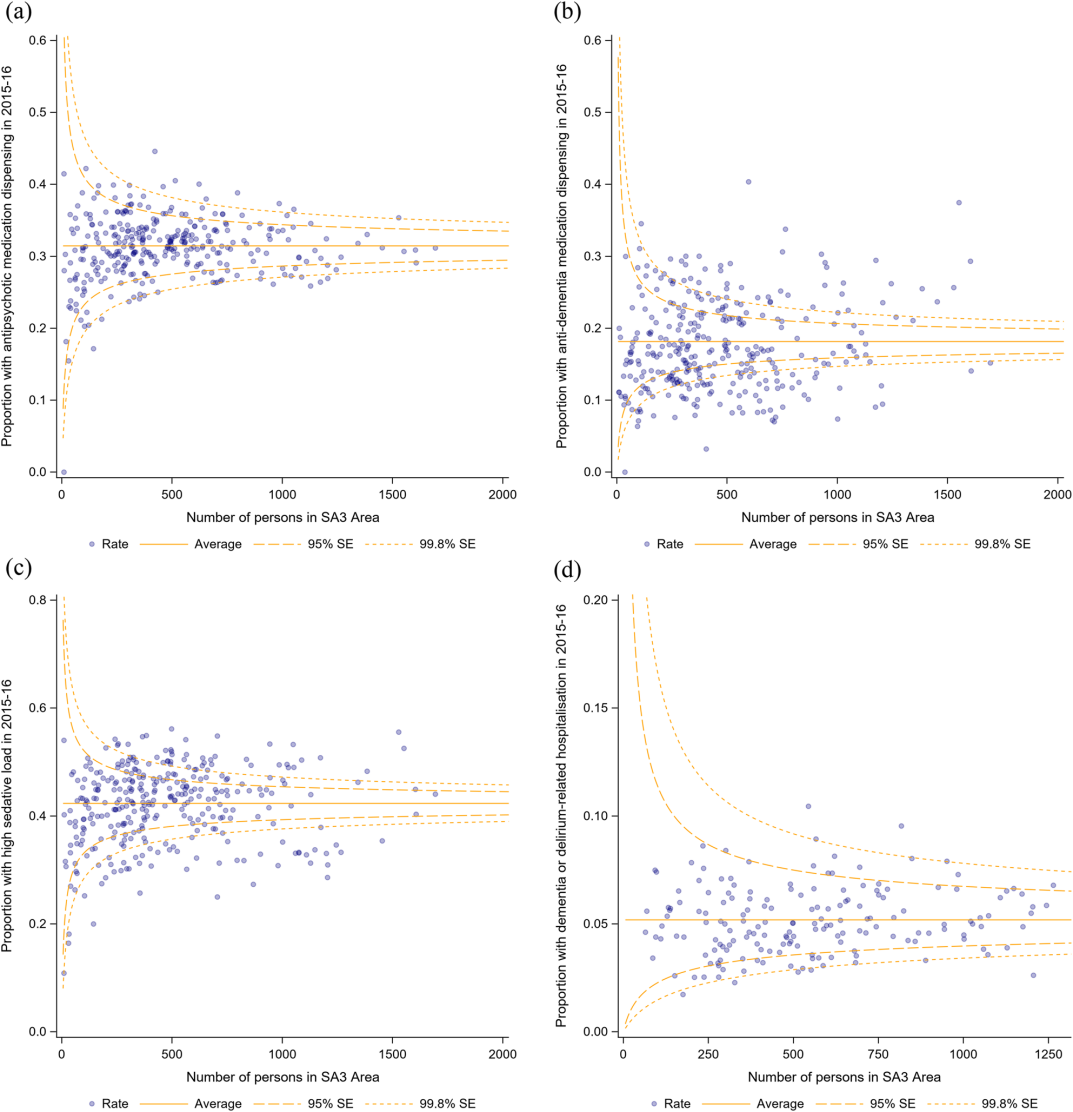
Adjusted geographical variation in (**a**) proportion of aged care users with dementia who were dispensed an antipsychotic medication in 2015/16; (**b**) proportion of aged care users with dementia who were dispensed a cholinesterase inhibitor or memantine in 2015/16; (**c**) proportion of aged care users with dementia with at least one 90-day period with high sedative medication load in 2015/16, and; (**d**) proportion of aged care users with dementia with a dementia or delirium-related hospitalisation in 2015/16 (New South Wales, Victoria, and South Australia only).

In the 2015/16 financial year, the incidence rate of antipsychotic medicine dispensing was lower among women than men and with increasing age, but higher among those born outside Australia, living outside metropolitan areas, and with more comorbidities (Table 3). The incidence rate of antipsychotic medicine dispensing varied across states (Table 4). Of 331 geographical areas across Australia, the proportion of people with dementia who were dispensed an antipsychotic medicine ranged from 0 to 44.7% (interquartile range (IQR) = 28.9–33.8%). There were 34 (1.6%) areas where the proportion was above the upper 95%CI around the population mean, and 41 (1.9%) where the proportion was below the lower 95%CI (Fig. 1).

**Table 3:** Factors associated with clinical quality indicator performance, July 2015–June 2016.

	aIRR (95%CI)			
	Antipsychotic medicine dispensing (*n* = 159,378)	Cholinesterase inhibitor and memantine dispensing (*n* = 159,378)^a^	High sedative load (*n* = 159,378)	Dementia or delirium-related hospitalisations^b^ (*n* = 107,804)
Age 1 July 2015^c^	0.85 (0.83–0.87)	0.96 (0.93–0.98)	0.97 (0.96–0.98)	0.62 (0.59–0.66)
Female	0.87 (0.86–0.88)	0.71 (0.70–0.73)	1.00 (0.98–1.02)^d^	0.77 (0.74–0.80)
Born outside Australia	1.07 (1.05–1.09)	0.93 (0.91–0.95)	0.97 (0.95–0.98)	1.21 (1.14–1.28)
Regional/remote	1.05 (1.03–1.07)	0.88 (0.86–0.90)	1.01 (1.00–1.03)^d^	0.89 (0.84–0.95)
Number of comorbid conditions	1.01 (1.01–1.02)	0.98 (0.97–0.99)	1.02 (1.01–1.03)	1.01 (1.00–1.03)
**State**				
New South Wales (ref)	–	–	–	–
Victoria	1.11 (1.09–1.14)	1.32 (1.28–1.36)	1.28 (1.26–1.31)	1.03 (0.97–1.09) ^e^
Queensland	1.03 (1.01–1.06) e	1.01 (0.97–1.04)^d^	1.20 (1.17–1.22)	–
Western Australia	0.94 (0.91–0.97)	0.94 (0.90–0.88)	1.14 (1.11–1.17)	–
South Australia	1.03 (1.00–1.07)^d^	1.13 (1.08–1.18)	1.32 (1.28–1.36)	0.91 (0.88–1.00) ^e^
Tasmania	0.98 (0.92–1.05)^d^	0.66 (0.59–0.73)	1.14 (1.08–1.21)	–
Northern Territory	0.73 (0.58–0.92) ^e^	0.97 (0.74–1.26)^d^	0.73 (0.58–0.91)^e^	–
Australian Capital Territory	0.87 (0.79–0.95) ^e^	1.59 (1.46–1.74)	0.90 (0.93–0.98)^d^	–

*aIRR* Adjusted incidence rate ratio, *CI* Confidence interval.

^a^Adjusted for time since cohort entry.

^b^New South Wales, Victoria, and South Australia only.

^c^Scaled up to 10-year increments.

^d^*p* > 0.05 after correction for multiple hypothesis testing.

^f^*p* between .0125 and .05, not considered statistically significant after correction for multiple hypotheses testing.

**Table 4:** Adjusted incidence rate of clinical quality indicator performance by state, 2015–2016.

	aIR per 1000 person/days (95%CI)				
	Antipsychotic medicine dispensing ^a^ (*n* = 159,378)	Cholinesterase inhibitor and memantine dispensing ^b^ (*n* = 159,378)	High sedative load ^a^ (*n* = 159,378)		Dementia or delirium-related hospitalisations ^a, c^ (*n* = 107,804)
New South Wales	1.06 (1.05–1.08)	0.65 (0.63–0.66)	1.25 (1.23–1.27)		0.18 (0.17–0.19)
Victoria	1.18 (1.16–1.20)	0.86 (0.84–0.88)	1.62 (1.60–1.64)		0.19 (0.18–0.20)
Queensland	1.10 (1.08–1.12)	0.65 (0.63–0.67)	1.53 (1.50–1.56)		–
Western Australia	1.00 (0.97–1.03)	0.55 (0.53–0.57)	1.42 (1.38–1.45)		–
South Australia	1.10 (1.06–1.13)	0.74 (0.71–0.77)	1.67 (1.63–1.71)		0.17 (0.15–0.18)
Tasmania	1.08 (1.02–1.14)	0.39 (0.36–0.44)	1.47 (1.39–1.54)		–
Northern Territory	0.77 (0.62–0.94)	0.57 (0.45–0.73)	0.87 (0.72–1.07)		–
Australian Capital Territory	0.92 (0.84–1.01)	1.05 (0.96–1.14)	1.10 (1.02–1.19)		–

*aIR* Adjusted incidence rate, *CI* Confidence interval.

^a^Adjusted for sex and age at 1 July 2015.

^b^Adjusted for sex, age at 1 July 2015, and time since cohort entry.

^c^New South Wales, Victoria, and South Australia only.

The 2015/16 incident rate for dispensing of cholinesterase inhibitors and memantine was lower with increasing age, for women, those born outside Australia, living outside metropolitan areas and with more comorbidities. These trends remained after removal of memantine (Supplementary Table A3). Geographically, the proportion of people with dementia who were dispensed an anti-dementia medicine ranged from 0 to 41.1% (IQR = 13.3–23.1%); in 85 (4.0%) geographical areas this proportion was above the upper 95%CI around the mean and 110 (5.2%) areas were below the lower 95%CI (Fig. 1). Similar geographic variations were identified after the removal of memantine (Supplementary Table A4, Supplementary Fig. A1).

The incidence rate of exposure to high sedative load in 2015/16 was not affected by sex or living in a regional area, but was lower with increasing age, among those born outside Australia and higher with more comorbidities. The proportion of people with dementia exposed to high sedative load in a geographical area from 0 to 56.1% (IQR = 37.1–47.0%); 80 (3.8%) areas recorded a higher proportion than the upper 95% CI around the mean and 74 (3.5%) recorded a lower proportion than the lower 95%CI (Fig. 1).

Among aged care users in New South Wales, Victoria, and South Australia, the 2015/16 incidence rate of dementia or delirium-related hospitalisations was lower among women than men, with increasing age, and in regional and remote areas. There were no significant differences in incidence rate between the three states. The proportion of people with dementia with a hospitalisation ranged from 0 to 10.4% (IQR = 4.1–6.1%) with 14 regions (0.7%) above the upper 95%CI around the mean and 31 regions (1.5%) below the lower 95%CI (Fig. 1).

## Discussion

There was little change in the incidence rate of four indicators of dementia care quality over five years among Australian aged care users with dementia. This has important implications for wellbeing because performance on these measures can impact quality of life, institutionalisation, and mortality for people with dementia^1–3^. Geographical variations in dementia CQIs like those reported here can guide targeted quality improvement efforts to where they are needed most.

The overuse of antipsychotic and other sedating medicines to manage behavioural symptoms for people with dementia has drawn major criticism internationally and in Australia in particular where a Royal Commission into Aged Care Quality and Safety has recently occured. Over- or inappropriate use of these medicines has serious associated risks of harm including permanent extra-pyramidal side effects, stroke, and mortality^21^. They also have limited efficacy over time^20^. As such, guidelines recommend that these medicines are only prescribed after non-pharmacological strategies have failed, at the lowest possible dose, when behavioural symptoms are severe, with written consent, and for the shortest possible timeframe. Despite efforts to reduce the prescribing of antipsychotic and other sedating medications, our results show that a third of Australian aged care users with dementia are dispensed an antipsychotic medicine each year with little decline in incidence and proportion of days exposed over time. Exposure to a high sedative load steadily increased over our study period, reaching 42% in 2016.

These rates are higher than reported in previous studies that included older people without dementia^41^ and are consistent with evidence from veteran populations where a modest decrease in dispensing over time has been reported^42^. There was also little variation between geographical regions, suggesting that high rates of antipsychotic and other sedative medicine prescribing are common across most regions of Australia. These results underscore the need for new organisational, regulatory, and clinical approaches to promoting deprescribing. Existing strategies, including tightened eligibility for medicine subsidising, appear to be insufficient even though deprescribing can be achieved for most people with dementia without an associated recurrence of behavioural symptoms^23^.

There has similarly been investment in community nursing and allied health services, hospital avoidance programs, and behaviour management programs in Australia to reduce the incidence of dementia and/or delirium-related hospitalisations^43^. These hospitalisations are indicators of potentially preventable complications like behavioural symptoms, infections, and falls in people with dementia^44^. Despite these investments, there has been a slight increase in the incidence rate of hospitalisations over time with little variation between geographical regions. As the overuse of sedating medications is a key contributor to potentially avoidable hospitalisations in people with dementia^45^, renewed efforts to promote deprescribing may have positive flow-on effects for hospital avoidance.

Conversely to the other indicators examined here, there is a recognised need to *increase* the incidence of prescribing of cholinesterase inhibitors and memantine^25^. Less than 20% of our cohort were dispensed a cholinesterase inhibitor or memantine over the study period, with large variation in the rates of dispensing across geographical regions. Clinical practice guidelines recommend that these medicines are trialled for all people with mild to moderate AD, while memantine is indicated for moderate to severe AD and may reduce the incidence of behavioural symptoms^25^. Given that an estimated 60–80% of dementias are caused by AD, our results are consistent with previous studies concluding that rates of cholinesterase inhibitor and memantine prescribing are lower than recommended and not improving with time^46, 47^. Our results also suggest that prescribing behaviour varies geographically, consistent with evidence that the professional training and culture in which a prescriber works influences their practice^48^. Access to medications that may delay cognitive decline is therefore inequitable for people with dementia.

Contributors to this problem may include that he efficacy of these medicines is variable and benefits appear to be time-limited, prompting conflicting clinical guidelines and indications between countries and over time^49^. Side effects can include gastrointensinal and cardiovascular symptoms. Variable attitudes about the value of these medicines have been reported among prescribers^28, 29^, particularly in the presence of side effects and polypharmacy^29^. Our data suggest that more work is needed to promote a shared understanding of when and how to use these medications so that prescribing rates increase. Recently published clinical guidelines for the deprescribing of anti-dementia medicines may prompt more cohesive prescribing practices^50^.

### Strengths and limitations

Strengths of this work include the large and national cohort of all older people with a recorded diagnosis of dementia who have access aged care services. The use of linked datasets means that there is limited loss to follow up. Our results demonstrate the potential value of routinely-collected integrated aged and health care data to assist with monitoring and benchmarking of dementia care quality. The ROSA cohort of older people with dementia can contribute to care monitoring on an ongoing basis because an average of 37,661 new cases of dementia are identified in aged care assessment data each year^15^. Many of these may not be identified via other sources.

There are important limitations to this work. The study cohort only includes people who have accessed government-subsidised aged care services, with a formal clinical diagnosis. One-half of men and one-third of Australian women never use an aged care service^51^, so are not included here. It is estimated that approximately half of people with dementia receive a formal diagnosis^52^ and those who do tend to have higher levels of education, be married, and live in metropolitan areas^53^. These factors impact the quality of a care a person with dementia receives^34^.

Entry to the study cohort occurred when the person was first recorded with dementia on an aged care assessment, and this may occur sometime after symptom onset and formal diagnosis. Nearly 33% of cases included here entered the cohort after they transitioned to permanent residential aged care, likely reflective of those with more severe or advanced dementia status. As such, there are likely to be gaps in our results regarding the quality of early clinical care. This is a problem particularly for monitoring of cholinesterase inhibitors and memantine, which are indicated only for mild to moderate symptoms. We were also unable stratify our results by dementia type, which is problematic as the type of dementia is known to impact the prevalence and importance of indicators measured here. For example, antipsychotic prescribing is particularly dangerous for those with vascular dementia and dementia secondarily to Parkinson’s disease where side effects are most risky^18^, and cholinesterase inhibitors are only indicated for Alzheimer’s disease^27^. Future work aiming to link ROSA data to the Australian Dementia Network CQR currently in development will help to overcome these limitations, as enrolment will occur at the point of diagnosis with more clinical detail available for monitoring^17^.

There are important limitations of using linked data that is not collected for research purposes. These include that medicine dispensing records do not include a reason for dispensing, so we are not able to comment on the appropriateness of the prescribing. In particular, our data regarding rates of cholinesterase inhibitor and memantine dispensing may also capture cases of inadequate deprescribing when these medications are no longer of benefit, which is common^54^. This muddies efforts to monitor efforts to increase appropriate prescribing rates over time. Our data also does not include privately-funded medicine dispensing, which accounts for a small minority of dispensing among older Australians. Data are de-identified, limiting the extent to which data about geographical variation in indicator performance can be used to target interventions. Linkage updates can be slow, complex, and expensive^55^, limiting access to contemporary data. There are also many factors that may impact care quality that are not captured within ROSA, including provider type and the availability of informal support^34^. Finally, we were only able to include hospital admission data for publicly-funded hospitals in South Australia, New South Wales, and Victoria in the current study.

## Conclusions and implications

Despite efforts to increase prescribing of cholinesterase inhibitors and memantine and decrease antipsychotic medication prescribing, sedative medication prescribing, and dementia-related hospitalisations, there has been little change in these four indicators of dementia care quality in Australian aged care users over time. Existing strategies to improve national performance on these indicators appear to be insufficient, even with the significant impact of these indicators on outcomes for people with dementia. In addition, marked variability in the use of cholinesterase inhibitors and memantine indicates a need for an agreed understanding of how and when these medicines should be used. These data demonstrate ways in which integrated aged and health care data sources can contribute to the monitoring of dementia care quality.

## Supplementary information

Supplementary Information.

## Data Availability

The data included in this research are available upon request to the authors.
