# Supplementary material for: Using data linkage for national surveillance of clinical quality indicators for dementia care among Australian aged care users
Source: Sci Rep. 2021 May 21;11:10674. doi: 10.1038/s41598-021-89646-x (PMC8140144; doi:10.1038/s41598-021-89646-x)
Supplement: Supplementary file 1 — Supplementary Information. [file 41598_2021_89646_MOESM1_ESM.docx]

# Title

Using data linkage for national surveillance of clinical quality indicators for dementia care among Australian aged care users

# Authors

Monica Cations, PhD^a,b*^, Catherine Lang, BSc(Hons)^a^, Stephanie A Ward, PhD^c,d,e^, Gillian E. Caughey PhD^a,f,g^, Maria Crotty, PhD^b,h^, Craig Whitehead, PhD ^b,h^, Susannah Ahern PhD^e^, John Maddison, PhD^i^, Maria C. Inacio PhD^a,g^

^a^Registry of Senior Australians, South Australian Health and Medical Research Institute, Adelaide SA, Australia

^b^College of Medicine and Public Health, Flinders University, Adelaide SA, Australia

^c^Centre for Healthy Brain Ageing, University of New South Wales, Sydney NSW, Australia

^d^Department of Geriatric Medicine, The Prince of Wales Hospital, Sydney NSW, Australia

^e^School of Public Health and Preventative Medicine, Monash University, Melbourne VIC, Australia

^f^Faculty of Health and Medical Sciences, University of Adelaide, Adelaide SA, Australia

^g^Division of Health Sciences, University of South Australia, Adelaide SA, Australia

^h^Southern Adelaide Local Health Network, SA Health, Adelaide SA, Australia

^i^Northern Adelaide Local Health Network, SA Health, Adelaide SA, Australia

***Corresponding author:** Dr Monica Cations, South Australian Health and Medical Research Institute, PO Box 11060, Adelaide SA, 5001, +618 8128 4236, [monica.cations@flinders.edu.au](mailto:monica.cations@flinders.edu.au)

**Supplementary Materials**

Table A1. Codes and datasets used to identify clinical quality indicators.

| **Description** | **Code** | **Dataset** |
| --- | --- | --- |
| **Antipsychotic medicines** ^a^ |  | Pharmaceutical Benefits Scheme |
| Chlorpromazine | N05AA01 |  |
| Fluphenazine | N05AB02^b^ |  |
| Trifluoperazine | N05AB06^b^ |  |
| Periciazine | N05AC01 |  |
| Haloperidol | N05AD01 |  |
| Ziprasidone | N05AE04 |  |
| Lurasidone | N05AE05 |  |
| Flupentixol | N05AF01 |  |
| Zuclopenthixol | N05AF05 |  |
| Clozapine | N05AH02 |  |
| Olanzapine | N05AH03 |  |
| Quetiapine | N05AH04 |  |
| Asenapine | N05AH05 |  |
| Amisulpride | N05AL05 |  |
| Risperidone | N05AX08 |  |
| Aripiprazole | N05AX12 |  |
| Paliperidone | N05AX13 |  |
| Brexpiprazole | N05AX16^b^ |  |
| **Cholinesterase inhibitors and memantine** ^a^ | | Pharmaceutical Benefits Scheme |
| Donepezil | N06DA02 |  |
| Galantamine | N06DA04 |  |
| Rivastigmine | N06DA03 |  |
| Memantine | N06DX01 |  |
| **Dementia and delirium-related hospitalisations ^c^** | | Hospital Admitted Data Collections ^d^ |
| Other symptoms and signs involving cognitive functions and awareness | R41 |  |
| Disorientation, unspecified | R410 |  |
| Other and unspecified symptoms and signs involving cognitive functions and awareness | R418 |  |
| Alzheimer’s disease | G30 |  |
| Lewy body disease | G31.3 |  |
| Dementia in Alzheimer’s disease | F00 |  |
| Vascular dementia | F01 |  |
| Dementia in other diseases classified elsewhere | F02 |  |
| Unspecified dementia | F03 |  |
| Delirium, not induced by alcohol and other psychoactive substances | F05 |  |

^a^ **Anatomical Therapeutic Chemical Classification codes recorded at dispensing**

^b^ Fluphenazine and trifluoperazine are no longer listed on the PBS but were listed on the PBS in 2016; brexpiprazole is currently listed on the PBS but was not listed in 2016.

^c^ Principal ICD-10-AM codes recorded at discharge

^d^ Integrated South Australian Activity Collection; New South Wales Addition Patient Data Collection; Victorian Admitted Episodes Dataset

Table A2. Results of sensitivity analysis examining dispensing of cholinesterase inhibitors by year, after removal of memantine

|  | 2011/12 | 2012/13 | 2013/14 | 2014/15 | 2015/16 | Absolute change ^a^ | aIRR (95%CI) ^b^ | *p* ^c^ |
| --- | --- | --- | --- | --- | --- | --- | --- | --- |
| Denominator (*n*) | 153,575 | 158,041 | 161,306 | 163,948 | 159,378 |  |  |  |
| Person-days (*n*) | 43,178,253 | 44,459,569 | 45,634,657 | 46,272,751 | 46,510,686 |  |  |  |
| Medicine dispensing (*n*) | 26,876 | 27,696 | 28,908 | 30,210 | 28,561 |  |  |  |
| Cumulative incidence (95% CI) | 17.5 (17.3-17.7) | 17.5 (17.3-17.7) | 17.9 (17.7-18.1) | 18.4 (18.2-18.6) | 17.9 (17.7-18.1) | 0.4 |  |  |
| Incidence per 1000p/d (95% CI) | 0.62 (0.62-0.63) | 0.62 (0.62-0.63) | 0.66 (0.65-0.66) | 0.65 (0.65-0.66) | 0.62 (0.61-0.62) | 0.00 | 1.01 (0.99-1.04) | 0.16 |

aIRR=Adjusted incidence rate ratio; CI=Confidence interval; p/d=Person-days

^a^ From 2011/2012 to 2015/2016 – crude difference only

^b^ Adjusted for age, sex, and time since cohort entry

^c^ Bonferroni correction applied

Table A3. Factors associated with cholinesterase inhibitor dispensing after removal of memantine, 2015-2016

|  | aIRR (95%CI) |
| --- | --- |
|  | Antidementia medicine dispensing  (*n*=159,378) ^a^ |
| Age at cohort entry ^b^ | 0.71 (0.70-0.72) |
| Female | 0.96 (0.94-0.98) |
| Born outside Australia | 0.90 (0.87-0.92) |
| Regional/remote | 0.90 (0.88-0.93) |
| Number of co-morbid conditions | 0.99 (0.98-0.99) |
| State |  |
| New South Wales (ref) | - |
| Victoria | 1.36 (1.32-1.40) |
| Queensland | 1.04 (1.00-1.08) ^d^ |
| Western Australia | 0.88 (0.84-0.92) |
| South Australia | 1.21 (1.16-1.26) |
| Tasmania | 0.70 (0.63-0.77) |
| Northern Territory | 0.99 (0.96-1.30) ^d^ |
| Australian Capital Territory | 1.63 (1.49-1.78) |

aIRR=Adjusted incidence rate ratio; CI=Confidence interval

^a^ Adjusted for time since cohort entry

^b^ Scaled up to 10-year increments

^d^ *p*>0.05 after correction for multiple hypothesis testing

Table A4. Adjusted incidence rate of cholinesterase inhibitor dispensing per 1000 person-days by state (*n*=159,378) after removal of memantine, 2015-2016

|  | aIR (95%CI) |
| --- | --- |
|  | Antidementia medicine dispensing ^b^ |
| New South Wales | 0.60 (0.59-0.62) |
| Victoria | 0.82 (0.80-0.94) |
| Queensland | 0.63 (0.61-0.65) |
| Western Australia | 0.53 (0.51-0.56) |
| South Australia | 0.73 (0.80-0.76) |
| Tasmania | 0.40 (0.36-0.44) |
| Northern Territory | 0.55 (0.53-0.71) |
| Australian Capital Territory | 0.99 (0.90-1.08) |

aIR=Adjusted incidence rate; CI=Confidence interval

^b^ Adjusted for sex, age and time since cohort entry

| (a)  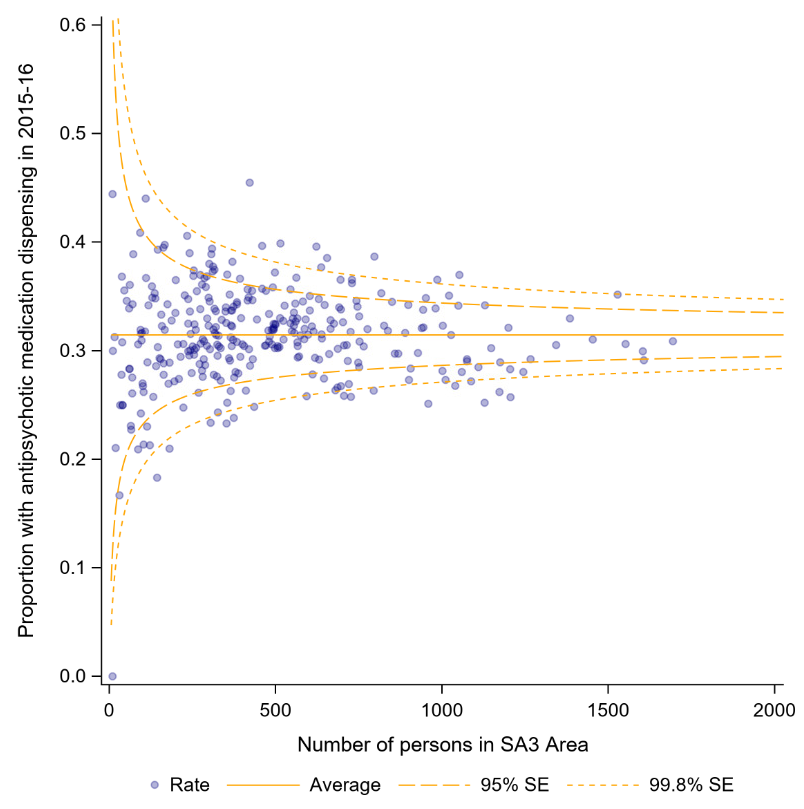 | (b) 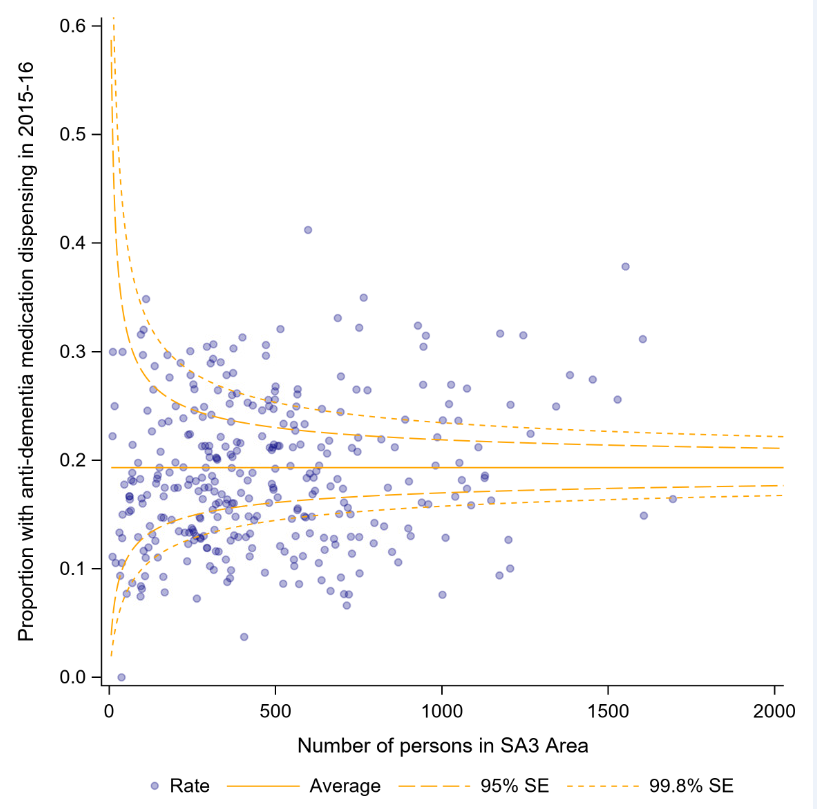 |
| --- | --- |
| (c) 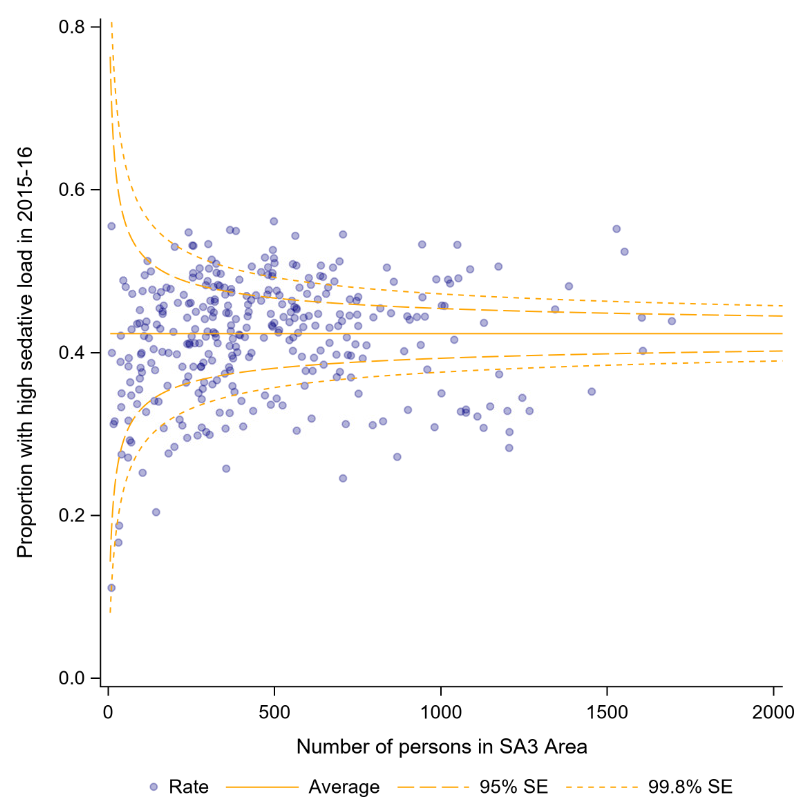 | (d) 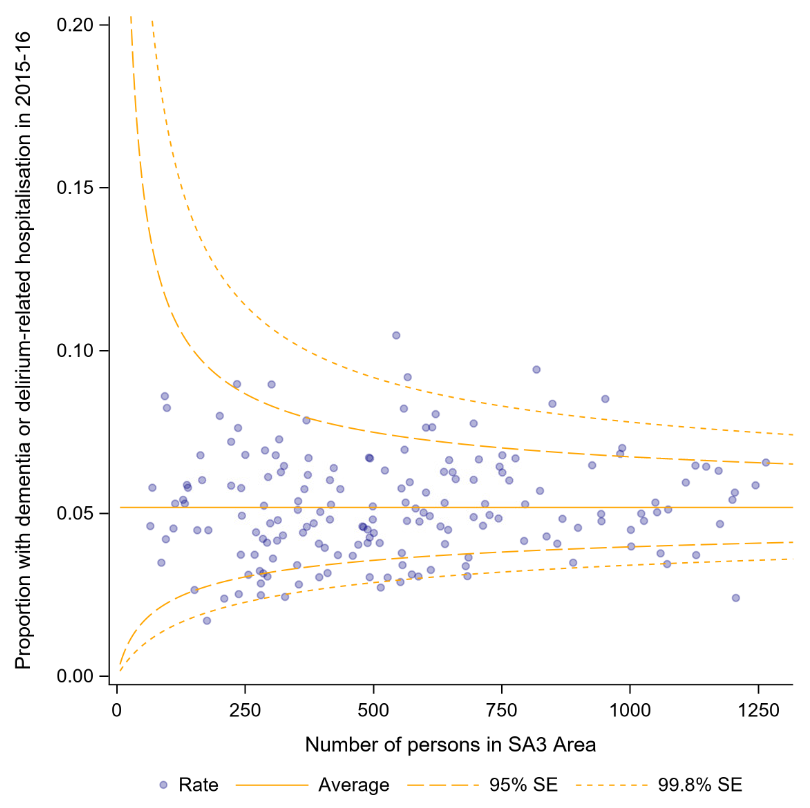 |
| Figure A1. Crude geographical variation in (a) proportion of aged care users with dementia who were dispensed an antipsychotic medication in 2015/16; (b) proportion of aged care users with dementia who were dispensed a cholinesterase inhibitor or memantine in 2015/16; (c) proportion of aged care users with dementia with at least one 90-day period with high sedative medication load in 2015/16, and; (d) proportion of aged care users with dementia with a dementia or delirium-related hospitalisation in 2015/16 (New South Wales, Victoria, South Australia only). Figures generated using SAS Software, Version 9.4. Copyright 2015 SAS Institute Inc. SAS and all other SAS Institute Inc. product or service names are registered trademarks or trademarks of SAS Institute Inc., Cary, NC, USA. | |

| (a)  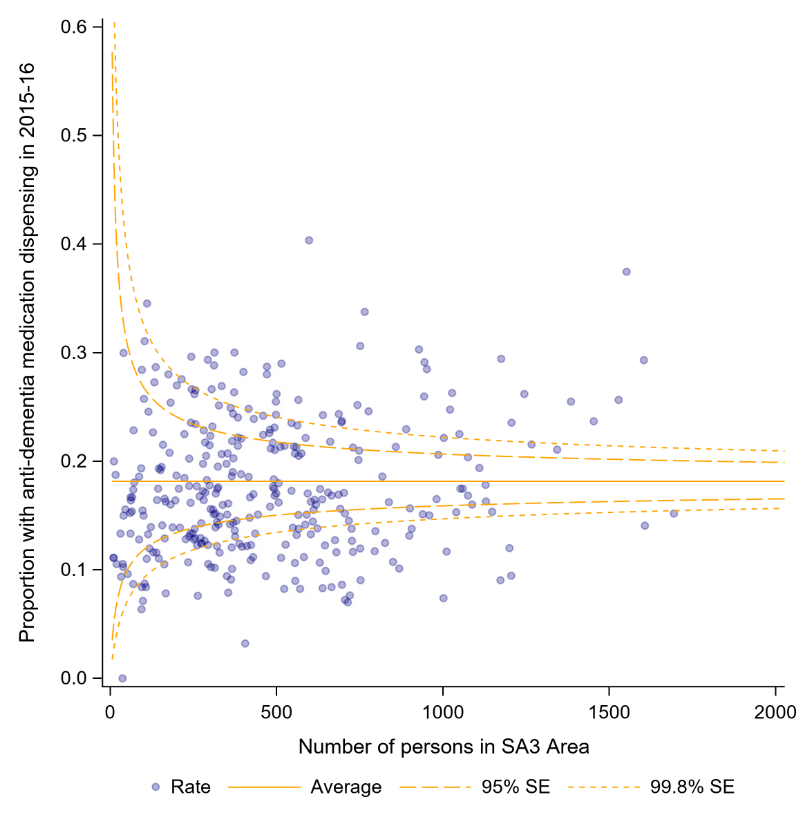 | (b)  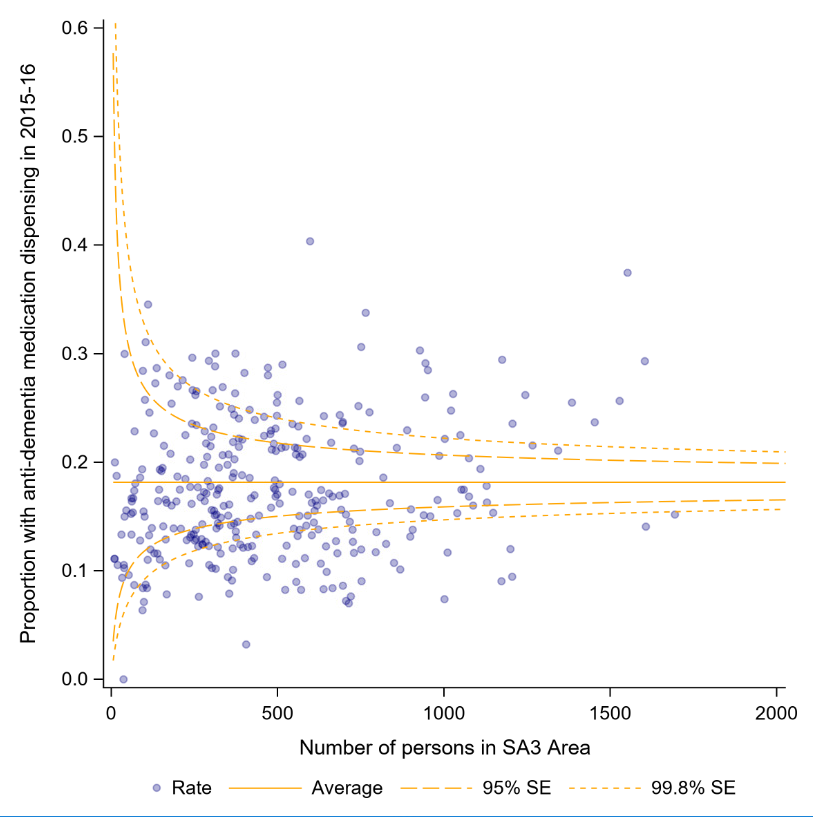 |
| --- | --- |

Figure S2. (a) Crude and (b) adjusted geographical variation proportion of aged care users with dementia who were dispensed a cholinesterase inhibitor in 2015/16, after removal of memantine Figures generated using SAS Software, Version 9.4. Copyright 2015 SAS Institute Inc. SAS and all other SAS Institute Inc. product or service names are registered trademarks or trademarks of SAS Institute Inc., Cary, NC, USA.
